# Serum albumin level for prediction of all-cause mortality in acute coronary syndrome patients: a meta-analysis

**DOI:** 10.1042/BSR20190881

**Published:** 2020-01-06

**Authors:** Lingjun Zhu, Miaomiao Chen, Xiaoping Lin

**Affiliations:** 1 Department of Cardiology, The Second Affiliated Hospital of Zhejiang University School of Medicine, Hangzhou 310009, Zhejiang, China; 2 Department of Ultrasound, The Second Affiliated Hospital of Zhejiang University School of Medicine, Hangzhou 310009, Zhejiang, China

**Keywords:** acute coronary syndrome, albumin, all-cause mortality, meta-analysis

## Abstract

The prognostic utility of serum albumin level as a predictor of survival in patients with acute coronary syndrome (ACS) has attracted considerable attention. This meta-analysis sought to investigate the prognostic value of serum albumin level for predicting all-cause mortality in ACS patients. A systematic literature search was conducted in Pubmed and Embase databases until 5 March 2019. Epidemiological studies investigating the association between serum albumin level and all-cause mortality risk in ACS patients were included. Eight studies comprising 21667 ACS patients were included. Meta-analysis indicated that ACS patients with low serum albumin level had an increased risk of all-cause mortality (risk ratio [RR] 2.15; 95% confidence interval [CI] 1.68–2.75) after adjusting for important covariates. Subgroup analysis showed that the impact of low serum albumin level was stronger in hospital mortality (RR 3.09; 95% CI 1.70–5.61) than long-term all-cause mortality (RR 1.75; 95% CI 1.54–1.98). This meta-analysis demonstrates that low serum albumin level is a powerful predictor of all-cause mortality in ACS patients, even after adjusting usual confounding factors. However, there is lack of clinical trials to demonstrate that correcting serum albumin level by means of intravenous infusion reduces the excess risk of death in ACS patients.

## Introduction

Acute coronary syndrome (ACS) is a complex heterogeneous clinical syndrome including unstable angina, non-ST elevation myocardial infarction (NSTEMI), and ST elevation myocardial infarction (STEMI) [1]. Despite the great advancement in medical care, ACS remains a leading cause of considerable morbidity and mortality [2]. Accurate prediction of adverse prognosis is essential for better management of ACS. Therefore, identification of potential prognostic factors is required for improving the prognosis of these patients.

Albumin is a predominant protein in human plasma [3]. Normal serum albumin level fluctuates from 3.5 to 5.0 g/dl in adults. Serum level of albumin less than 3.5 g/dl is usually defined as hypoalbuminemia. Hypoalbuminemia is predominantly caused by malnutrition, inflammation or cachexia [4]. Low serum albumin level has been identified as a risk factor for the development of coronary artery disease [5]. Several epidemiological studies [6–13] have examined the association between serum albumin and adverse outcomes in ACS patients. However, these available studies differed from study design, population studied, sample size, cut-off level of low serum albumin, and adjustment for covariates. To the best of our knowledge, no previous meta-analysis has addressed this issue. We therefore conducted this meta-analysis of the available literature to evaluate the prognostic role of low serum albumin level in terms of in-hospital and long-term all-cause mortality in patients with ACS.

## Materials and methods

### Literature search

The current meta-analysis followed the guideline of Meta-Analysis of Observational Studies in Epidemiology [14]. We conducted a comprehensive literature search through Pubmed and Embase databases until 5 March 2019. The following keywords were used to identify eligible studies: ‘hypoalbuminemia’ OR ‘albumin’ AND ‘acute coronary syndrome’ OR ‘unstable angina’ OR ‘acute myocardial infarction’ AND ‘mortality’ OR ‘death’. References of eligible studies and related reviews were manually screened to identify possible missing articles.

### Study selection

Included studies had to meet all the following inclusion criteria: (1) observational studies enrolling patients with ACS; (2) hypoalbuminemia or low albumin level as exposure; (3) all-cause mortality as outcome of interest; and (4) reported most fully adjusted risk ratio (RR) or odds ratio (OR) with their 95% confidence interval (CI) of in-hospital mortality and follow-up all-cause mortality. The following exclusion criteria were applied: (1) meeting abstracts and (2) outcome reported by continuous albumin level.

### Data extraction and quality assessment

Relevant data were independently extracted by two authors using a standardized form. The following data were obtained from each study: last name of the first author, year of publication, country of origin, subtype of patients, sample size, proportion of men, mean and range of age, duration of follow-up, categories of low albumin level, number of death events, most fully adjusted RR with 95% CI, variable controlled in the multivariable model. The Newcastle–Ottawa scale (NOS) for cohort studies was used to examine the methodological quality of included studies [15]. Studies with a score ≥7 were deemed as high quality. Disagreements in data extraction and quality assessment were resolved by discussion.

### Statistical analysis

All statistical analyses were conducted using Stata 12.0 software (Stata Corporation, College Station, TX, U.S.A.). Statistical heterogeneity was checked by the Cochran’s Q test and *I^2^* statistics. We selected a random-effect model when the *I^2^* statistics ≥ 50% or *P*<0.10 of the Cochran Q test; otherwise, a fixed-effect model was used for the meta-analysis. To examine the impact of individual studies on the overall risk estimate, we performed a sensitivity analysis by omitting any single study at each time. Subgroup analyses were conducted according to study design (retrospective *vs.* consecutive), country (China *vs.* others), sample size (≥1000 *vs.* <1000), type of patients (all ACS *vs.* acute myocardial infarction (AMI)), follow-up duration (in-hospital *vs.* ≥1 year), cut-off value of low albumin level (single *vs.* tertiles/quartile), and NOS points (≥7 *vs. <*7). Begg’s rank correlation [16] and Egger’s linear regression test [17] were applied to examine the publication bias. In the presence of publication bias, a trim-and-fill method was used to explore the impact of publication bias.

## Results

### Search results and study characteristics

**Figure 1:**
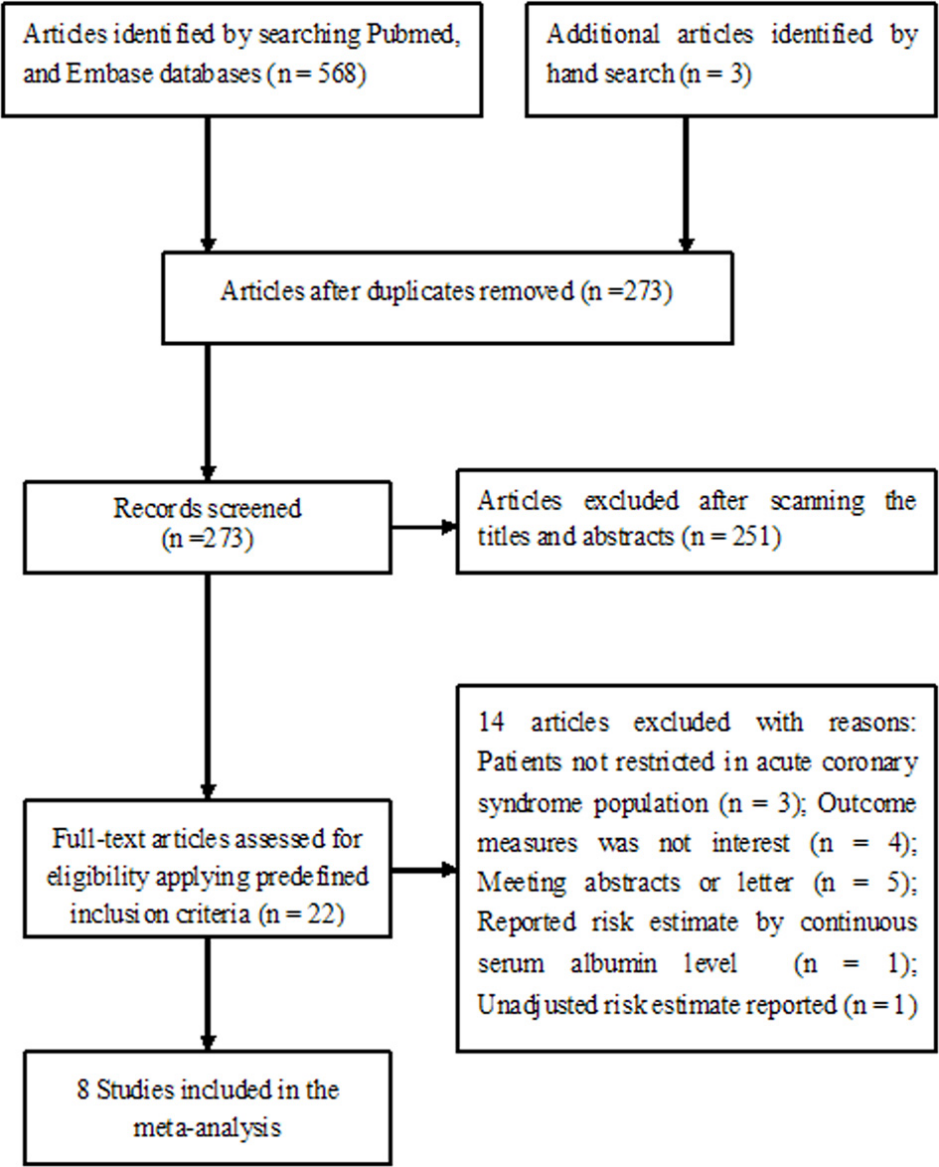
Flow chart of studies selection process

Our electronic literature search obtained 568 potentially relevant articles. Three additional articles were identified by manual search. Of which, 298 duplicated records were excluded. After scanning the titles and abstracts, we removed 251 articles because they did not focus on the topic. During full-text assessment, 14 articles were further removed for various reasons. Finally, eight studies [6–13] were included in the meta-analysis (Figure 1).

Table 1 summarizes the main characteristics of eligible studies. These studies were published from 2006 to 2018 and conducted in China [6,12,13], Turkey [7,9], Israel [10], Japan [8], and Mexico [11]. The sample size of each study ranged from 62 to 8750, with a total of 21667 ACS patients. Three studies [8,9,13] were consecutive designs and others were retrospective studies. The duration of follow-up was up to 6.1 years. According to the quality assessment criteria, five studies [7,9–11,13] were grouped as high quality.

**Table 1:** Main characteristic of the included studies

Author, year	Country	Study design	Sample size (% male)	Type of patients	Age (years)	Comparison of albumin level	Outcome measures HR/OR (95% CI)	Follow-up duration	Adjustment for covariates	NOS score
Deng, 2006 [6]	China	Retrospective	82 (72.0)	AMI	65.4 ± 11.4	<3.5 *vs.* ≥3.5 g/dl	Total deaths: 10 4.17 (1.06–16.4)	In-hospital	Age, sex, DM, hypertension, dyslipidemia, previous MI, and hs-CRP	6
Oduncu, 2013 [7]	Turkey	Retrospective	1706 (75)	STEMI	61.3 ± 12.3	<3.5 *vs.* ≥3.5 g/dl	Total deaths: 214 2.98 (1.35–6.58)	3.5 years	Age, sex, DM, hypertension, dyslipidemia, COPD, previous MI or HF, PAD, BMI, hemoglobin, CRP,WBC, BNP,heart rate, TG, LDL, cardiogenic shock, intra-aortic balloon pump use, reperfusion time, eGFR, multivessel disease, TIMI, troponin I, LVEF, major bleeding, blood transfusion, and medications	8
Sujino, 2015 [8]	Japan	Consecutive	62 (58.1)	STEMI	88.1 ± 2.5	Hypoalbuminemia	Total deaths: 14 6.25 (1.14–34.3)	In-hospital	Multivariate analysis	6
Kurtul, 2015 [9]	Turkey	Consecutive	1303 (69.8)	ACS	61.2 ± 13.1	<3.65 *vs.* ≥3.65 g/dl	Total deaths: 49 4.33 (2.03–8.62)	In-hospital	Age, women, SBP, DM, active smoking, LVEF, BMI, eGFR, hematocrit, type of ACS, admission glucose, and hs-CRP	7
Plakht, 2016 [10]	Israel	Retrospective	8750 (70.8)	AMI	65 ± 14	≤3.4 *vs*. >4.1 g/dl	Total deaths: 2975 1.70 (1.48–1.95)	6.1 years	Multivariate analysis	7
González- Pacheco, 2017 [11]	Mexico	Retrospective cohort	7192 (80.2)	ACS	49–71	Quartile 1 vs.4; ≤3.5 *vs.* >4.08 g/dl	Total deaths: 310 1.88 (1.23–2.86)	In-hospital	Age, gender, Killip class, LVEF, SBP, renal dysfunction, WBC, hs-CRP, and heart rate	7
Wang, 2017 [12]	China	Retrospective cohort	267 (78.7)	STEMI	65.0 ± 12.2	≤3.5 *vs.* >3.5 g/dl	Total deaths: 41; 2.61 (1.17–5.85)	1.0 year	Age, BMI, SDP, BDP, TG, TC, lactate dehydrogenase, Killip class, hemoglobin, and creatinine	6
Xia, 2018 [13]	China	Consecutive	2305 (79.7)	AMI	Median 65	Tertile 1 vs. .3; ≤3.62 *vs.* >4.08 g/dl	Total deaths: 262; 1.74 (1.21–2.52)	3 years	Age, heart failure, DM, eGFR, PCI, triple-vessel coronary and left main artery disease	7

Abbreviations: BMI, body mass index; BNP, B-type natriuretic peptide; COPD, chronic obstructive pulmonary disease; DM, diabetes mellitus; eGFR, estimated glomerular filtration rate; HF, heart failure; HR, hazard ratio; hs-CRP, high-sensitivity C-reactive protein; LDL, low-density lipoprotein; LVEF, left ventricular ejection fraction; MI, myocardial infarction; PAD, peripheral arterial disease; PCI, percutaneous coronary intervention; SBP, systolic blood pressure; TC, total cholesterol; TG, triglyceride; TIMI, thrombolysis in myocardial infarction; WBC, white blood cell.

### All-cause mortality

**Figure 2:**
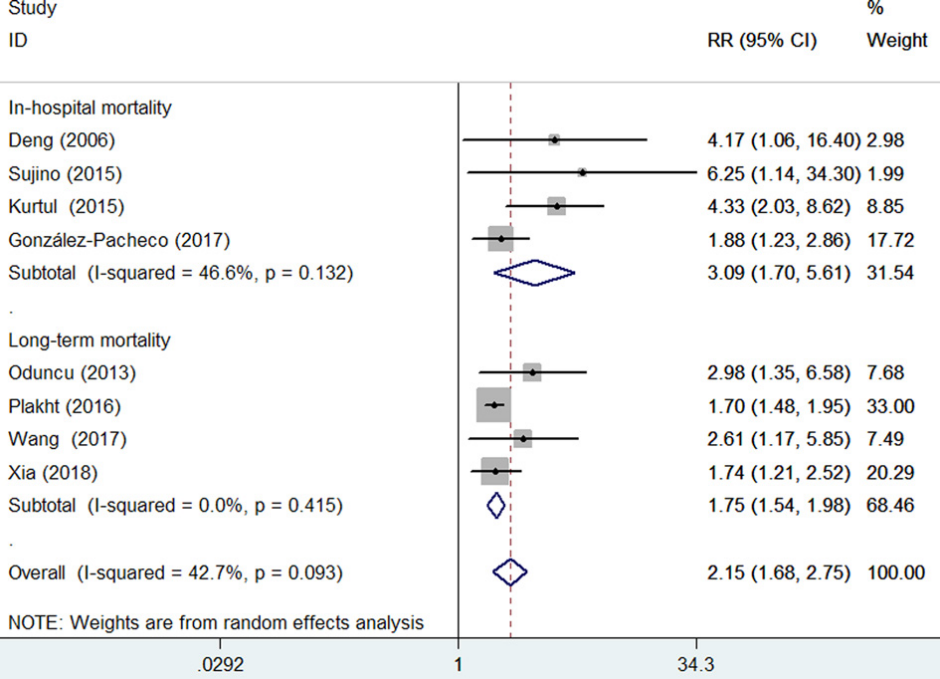
Forest plots showing pooled RR with 95% CI of all-cause mortality for the low versus reference normal serum albumin level

**Figure 3:**
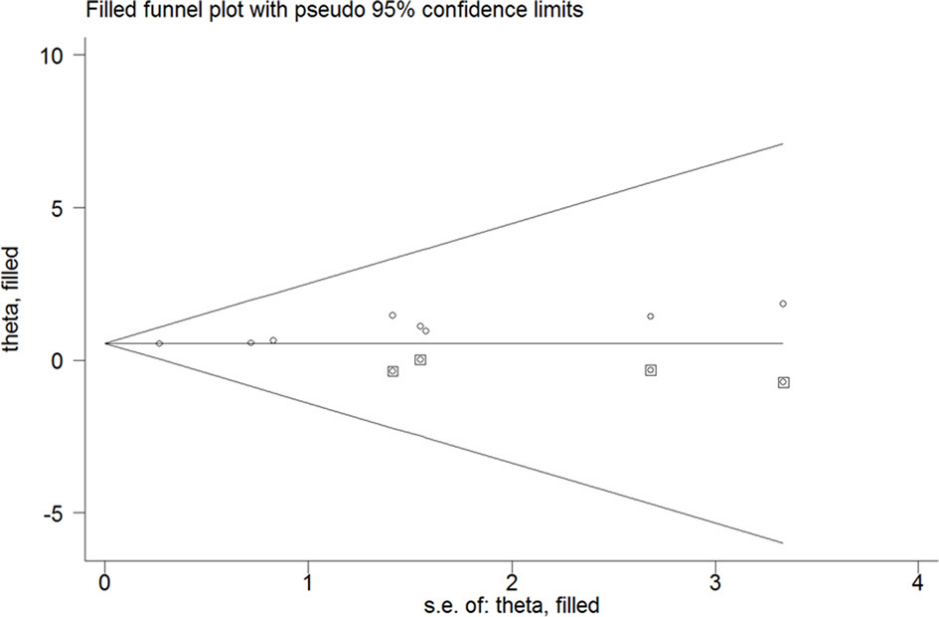
Funnel plot of low serum albumin ratio with all-cause mortality The circles alone are real studies and the circle enclosed in box is ‘filled’ study.

Four studies [6,8,9,11] reported the in-hospital mortality as an outcome and another four studies [7,10,12,13] focused on the long-term all-cause mortality. As shown in Figure 2, overall low serum albumin level was associated with an increased risk of all-cause mortality (RR 2.15; 95% CI 1.68–2.75) in a random-effect model, with substantial heterogeneity across studies (*I^2^* = 42.7%; *P*=0.093). Sensitivity analysis confirmed the robustness of the pooling results (data not shown). Potential publication bias was identified on the basis of Egger’s test (*P*=0.004) and Begg’s test (*P*=0.063). The trim-and-fill adjustment approach indicated four missing studies in the funnel plot (Figure 3). However, imputing these four potential missing studies did not significantly change the prognostic significance (RR 1.71; 95% CI 1.12–2.60; *P*=0*.*013) and displayed no substantial changes in the fixed-effect model. Results stratified by follow-up duration suggested a stronger risk of low serum albumin level on in-hospital mortality (RR 3.09; 95% CI 1.70–5.61) than long-term all-cause mortality (RR 1.75; 95% CI 1.54–1.98). In addition, the prognostic significance of low serum albumin level for all-cause mortality was observed in each subgroup (Table 2).

**Table 2:** Subgroup analysis for all-cause mortality

Subgroup	Number of studies	Pooled RR	95% CIs	Heterogeneity between studies
Study design				
Retrospective	5	1.85	1.54–2.22	*P*=0.346; *I^2^* = 10.5%
Consecutive	3	2.97	1.33–6.63	*P*=0.041; *I^2^* = 68.7%
Country				
China	3	1.97	1.40–2.77	*P*=0.357; *I^2^* = 2.8%
Others	5	2.33	1.60–3.40	*P*=0.041; *I^2^* = 59.9%
Sample size				
>1000	5	2.00	1.55–2.57	*P*=0.094; *I^2^* = 49.6%
<1000	3	3.28	1.72–6.23	*P*=0.613; *I^2^* = 0.0%
Type of patients				
All ACS	2	2.70	1.20–6.08	*P*=0.051; *I^2^* = 73.8%
AMI	6	1.93	1.53–2.43	*P*=0.260; *I^2^* = 23.2%
Study quality				
NOS ≥ 7	5	2.00	1.55–2.57	*P*=0.094; *I^2^* = 49.6%
NOS < 7	3	3.28	1.72–6.23	*P*=0.613; *I^2^* = 0.0%

## Discussion

The current meta-analysis demonstrates that low serum albumin level is a powerful predictor of all-cause mortality in ACS patients, even after adjusting usual confounding factors. ACS patients with hypoalbuminemia had a three-fold risk of in-hospital mortality and 75% higher risk of long-term all-cause mortality, respectively. Furthermore, the prognostic significance of low serum albumin level on all-cause mortality was consistent in each predefined subgroups. Our meta-analysis suggests that baseline serum albumin level may be used for risk prediction in patients with ACS.

Serum albumin level is a simple routine laboratory test. Given its anti-inflammatory, antioxidant, anticoagulant, and anti-platelet aggregation activity [18], serum albumin may contribute to the progression of coronary artery disease. Of ACS patients, low serum albumin level at admission was also an independent predictor of heart failure [7,11], extent and complexity of coronary artery disease [9], and contrast-induced acute kidney injury [19]. ACS patients represent a heterogeneous clinical group. Hypoalbuminemia was associated with angiographic no-reflow after primary percutaneous coronary intervention in patients with STEMI [20] and in-hospital adverse outcomes in non-ST elevation ACS [21]. Moreover, analysis of serum albumin level by continuous variables in patients with first-onset acute myocardial infarction, each 1 g/dl albumin level reduction exhibited a 66 and 47% higher risk of all-cause mortality and cardiovascular death, respectively [13]. Apart from ACS, hypoalbuminemia was possibly an independent predictor of in-hospital death and long-term all-cause mortality in patients with acute or chronic heart failure [22] and various cancers [23].

Increased inflammation has been linked to decreasing synthesis and increasing catabolism of albumin [24]. Serum albumin exerts anti-inflammatory function in physiological condition [25]. Inflammation may be an important confounding factor for the prognostic utility of serum albumin. Albumin can promote the formation of anti-inflammatory lipoxins, resolvins, and protectins [26]. Low albumin level can result in decreased formation of lipoxins, resolvins and protectins, tilts the balance more toward pro-inflammatory events, which lead to increased risk of death for the critically ill [27,28]. Moreover, hypoalbuminemia was linked with increased oxidative stress, platelet activation and aggregation, which triggered the thrombotic events [10]. However, whether hypoalbuminemia is just a biomarker or a risk factor in the course of ACS needs to be further clarified in future studies.

Several limitations of this meta-analysis should be noted. First, most of the included studies adopted the retrospective design, which may have an inherent risk of recall bias and selection bias. Second, publication bias was found according to the Begg’s test and Egger’s test. However, a trim-and-fill analysis revealed that the conclusion was not affected by potential publication bias. Third, not all included studies adjusted the systemic inflammatory markers such as C-reactive protein, which has been identified as an independent prognostic factor. Importantly, serum albumin level is also influenced by several clinical conditions other than nutritional status. Lack of adjustment for inflammatory cytokines, chronic kidney disease, liver function, and other unknown confounding factors might have led to overestimate risk estimate. Finally, our conclusion was established on a single determination of baseline serum albumin level. Dynamic measurements of serum albumin level may provide an additional prognostic significance.

In conclusion, low serum albumin level is a powerful predictor of all-cause mortality in ACS patients, even after adjusting usual confounding factors. However, there is lack of clinical trials to demonstrate that correcting serum albumin level by means of intravenous infusion reduces the excess risk of death in patients with ACS.

## Abbreviations

ACS: acute coronary syndrome
CI: confidence interval
NOS: Newcastle–Ottawa scale
RR: risk ratio
STEMI: ST elevation myocardial infarction
